# Predictive Factors of Inpatient Rehabilitation Outcomes and Stay: A Machine Learning Study with Temporal Validation

**DOI:** 10.3390/healthcare14142167

**Published:** 2026-07-17

**Authors:** Andrea Campagner, Claudio Cordani, Catia Pelosi, Lorenza Buttafava, Lucia Imperiali, Stefano Borghi, Dario Grippa, Carlotte Kiekens, Stefano Negrini, Giuseppe Banfi, Federico Pennestrì, the PREPARE Project Group

**Affiliations:** 1 Laboratory of Mechanics of Biological Structures, IRCCS Galeazzi-Sant’Ambrogio Hospital, Via Belgioioso 157, 20157 Milan, Italy; andrea.campagner@unimib.it; 2 Department of Informatics, Systems and Communications, University of Milan Bicocca, Via Sarca 336, 20126 Milan, Italy; 3 Laboratory of Evidence-Based Rehabilitation, IRCCS Galeazzi-Sant’Ambrogio Hospital, Via Belgioioso 157, 20157 Milan, Italy; claudio.cordani@grupposandonato.it (C.C.); carlotte.kiekens@isico.it (C.K.); stefano.negrini@unimi.it (S.N.); 4 Specialistic Rehabilitation, IRCCS Galeazzi-Sant’Ambrogio Hospital, Via Belgioioso 157, 20157 Milan, Italy; medicalcentersnc@gmail.com (C.P.); dario.grippa@grupposandonato.it (D.G.); 5 Clinical Epidemiology Unit, IRCCS Galeazzi-Sant’Ambrogio Hospital, Via Belgioioso 157, 20157 Milan, Italy; lorenzabuttafava@gmail.com; 6 Laboratory of Movement and Sport Science, IRCCS Galeazzi-Sant’Ambrogio Hospital, Via Belgioioso 157, 20157 Milan, Italy; lucia.imperiali@grupposandonato.it (L.I.); stefano.borghi@grupposandonato.it (S.B.); 7 Department of Biomedical, Surgical and Dental Sciences, University of Milan “La Statale”, Via Festa del Perdono 7, 20122 Milan, Italy; 8 Faculty of Medicine and Surgery, Vita-Salute San Raffaele University, Via Olgettina 58, 20132 Milan, Italy; banfi.giuseppe@hsr.it; 9 Scientific Direction, IRCCS Galeazzi-Sant’Ambrogio Hospital, Via Belgioioso 157, 20157 Milan, Italy

**Keywords:** discharge destination, functional outcomes, hip and knee osteoarthritis, inpatient rehabilitation, length of stay, machine learning, patient stratification

## Abstract

**Background/Objectives**: Despite the increasing rates of disability associated with aging, obesity, and osteoarthritis requiring surgery, optimizing rehabilitation after hospital discharge remains a major challenge and a key determinant of care safety, quality, and sustainability. The aim of this exploratory study is to evaluate the predictive performance of machine learning (ML) models for predicting Inpatient Rehabilitation Length Of Stay (IRLOS), Function in the Activities of Daily Living (FADL) and discharge destination (DD) in patients who underwent total joint replacement for hip and knee osteoarthritis, using Real-World Data routinely collected in a tertiary orthopedic hospital. **Methods**: 2103 patients were included and temporally split into a development cohort (2019; *n* = 1711) and a temporal validation cohort (2018; *n* = 392). A total of 73 routinely collected perioperative variables were used to train multiple ML models, including both black-box and transparent methods. IRLOS and FADL were modeled as regression tasks, while DD was treated as a binary classification task. Model development followed a rigorous pipeline with feature selection, cross-validation, and hyperparameter tuning. Performance was assessed using appropriate metrics and evaluated across joint type (hip/knee) and via temporal validation. Model interpretability was examined using SHAP and model-specific analyses, further supported by clinical analysis. **Results**: In temporal validation, models achieved modest performance for IRLOS (R^2^ = 0.17; MAE = 2.52 days) and FADL (R^2^ = 0.25; MAE = 2.25 days), with no significant performance degradation over time. DD prediction showed good discrimination (AUC = 0.85; balanced accuracy = 0.80) despite outcome imbalance, with high sensitivity (0.92) and negative predictive value (≈1.00), but low positive predictive value (0.09). Performance was stable across hip and knee subgroups. The interpretability analysis further highlighted several key predictors related to perioperative complexity (e.g., surgical duration and transfusion), baseline functional status, and social factors (e.g., living situation and employment), confirming that rehabilitation outcomes are multidimensional and influenced by both medical and non-medical determinants. **Conclusions**: The analysis identified several relevant predictors related to perioperative complexity, baseline functional status, and social context. The models showed stable behavior across intervention type and across time through temporal validation, suggesting that they capture relevant patterns in rehabilitation pathways, although predictive performance was moderate.

## 1. Introduction

In aging societies characterized by increasing healthcare demand, both in quantitative (e.g., number of patients) and in qualitative terms (e.g., patients affected by comorbidities, cognitive and motor limitations, and social isolation), ensuring safe care transitions is crucial to reduce hospital overload, waiting lists, avoidable readmissions, prolonged ward stay and minimize out-of-pocket expenditure [1,2,3,4,5,6,7,8]. Digitally collected RWD from comparable patient populations can support clinicians in estimating disease trajectories, integrating evidence with clinical experience, and communicating care options to patients and caregivers [9]. In this context, Computerized Clinical Decision Support Systems (CDSSs) [10] can combine routinely collected RWD to support prognostic assessment and individualized decision-making.

Previous studies have investigated predictors of rehabilitation outcomes after total joint replacement primarily using conventional statistical approaches, such as linear and logistic regression [11]. However, rehabilitation outcomes are likely influenced by complex interactions among demographic, clinical, functional and social factors [12,13], and it remains unclear whether machine-learning methods can exploit these relationships to improve prediction using routinely collected data [14,15]. Furthermore, evidence regarding the temporal robustness and generalizability of such models in rehabilitation settings remains limited [16].

The aim of this exploratory study is to evaluate the predictive performance and temporal generalizability of machine learning (ML) models for predicting Inpatient Rehabilitation Length Of Stay (IRLOS), Function in the Activities of Daily Living (FADL) and discharge destination (DD) in a population of patients who underwent total joint replacement for hip and knee osteoarthritis, a condition responsible for increasing disability in aging countries worldwide [17,18,19], using routinely collected real-world hospital data. IRCCS Ospedale Galeazzi-Sant’Ambrogio is a research hospital hub for hip and knee procedures in the Region of Lombardy, Italy, where supporting the continuity of hospital, intermediate, and primary care through systematic digitalization of infrastructure has become mandatory [20]. These predictors and models may contribute to the future development of CDSSs aimed at supporting clinicians in estimating rehabilitation trajectories and outcomes at the time of admission, provided validation before clinical implementation.

First, we describe data source and (a) patient population characteristics, in terms of diagnosis and procedure codes, hospital pathway, cohort size, cohort selection and outcome details (Section 2.1); (b) state-of-the-art ML methods, in terms of why they were chosen, how they were used, and what performance metrics were adopted (Section 2.2.). Then, model performance metrics are illustrated by task (IRLOS, FADL, and DD) and procedure (all, hip, knee) (Section 3). Finally, model performance and predictive values are discussed in light of the literature on the topic, clinical insight and research limitations (Discussion). Appendix A and Appendix B provide additional information on data preparation, model design, statistical analysis, stratified analysis, and interpretability analysis.

## 2. Materials and Methods

Linear and logistic regression techniques were used on a set of perioperative variables to evaluate the predictivity towards inpatient rehabilitation outcomes [21]. In this follow-up, we introduced and tested ML methodologies, a new inpatient rehabilitation outcome (FADL), and a totally new patient cohort for temporal validation. The ML methodology was adopted in compliance with the IJMEDI/CHAMAI checklist [22] for the reporting and evaluation of ML studies in healthcare.

### 2.1. Materials

Electronic hospital datasets were employed to identify all adult patients with hip or knee osteoarthritis who underwent total joint replacement and received specialist inpatient rehabilitation in another ward of the same hospital, in the same year. The population, comprising a total of 2103 patients, was stratified, according to a temporal criterion, into a development cohort (used for model training and internal validation) and a temporal validation cohort. In particular, the sub-cohort of patients who underwent surgery and subsequent inpatient rehabilitation between January and December 2019, larger in number (1711 patients), was employed to train the prediction models. By contrast, the sub-cohort of patients who underwent surgery and subsequent inpatient rehabilitation between January and December 2018, smaller in number (392 patients), was employed as the validation cohort. The temporal split was defined a priori before model development. The 2019 cohort was selected as the development dataset because it contained a larger number of observations, allowing more stable model training and hyperparameter optimization, whereas the 2018 cohort was retained as an independent temporally distinct hold-out sample.

The diagnoses and procedures of interest were identified based on the International Classification of Disease, 9th Revision, Clinical Modification (ICD-9-CM) codes 715.15, 715.16, 715.25 and 715.26 (diagnostic codes), 81.51 and 81.54 (procedure codes).

Once these patients were identified, clinical records data from the surgical and the specialist rehabilitation wards were retrieved. The internal inpatient identifier allowed tracking of the entire patient pathway from pre-operative surgical assessment to discharge. The collected dataset encompassed a total of 75 features. The data were collected between December 2023 and September 2025.

IRLOS was measured by the number of days spent in the specialized rehabilitation ward; DD was distinguished between home and institutional settings; FADL was measured by the Barthel Index of Autonomy in the ADL [23]. All outcomes were collected from the specialist rehabilitation unit hospital records.

Prior to analysis, both the development and validation cohorts’ data were pre-processed to ensure data quality and avoid bias in subsequent steps of the statistical analysis. See Appendix A, Data Preparation, for further details on pre-processing. The final dataset encompassed 1632 patients in the development cohort and 374 patients in the validation cohort, with 73 features (including the three target variables). Only variables that were routinely available at the time of admission to the rehabilitation ward were considered eligible predictors.

The complete list of included variables, together with information about their distribution statistics, is reported in Table 1.

### 2.2. Machine Learning and Statistical Methods

We considered three different variables as targets for the development of ML methods: IRLOS (as measured by the ‘Durata ricovero’ variable), DD (according to the ‘Outcome sociale’ variable), and FADL (as measured by the ‘BI.1’ variable). IRLOS and FADL were continuous outcomes; hence, they were analyzed using a regression approach, while DD was a categorical (binary) outcome. It was thus analyzed using a binary classification approach.

A variety of state-of-the-art ML methods for tabular data [24,25,26] were considered, including both black-box and transparent methods. For regression tasks (IRLOS and FADL), we considered the following classes of models: (regularized) linear regression (LiR) trained through stochastic gradient descent, decision tree (DT), support vector regression (SVM), random forest regressor (RF) and eXtreme gradient boosting (XGB). For the classification task (DD), we considered the following classes of models: (regularized) logistic regression (LoR), DT, SVM, RF and XGB. This modeling framework allows assessment of whether non-linear machine-learning approaches offer incremental predictive value over traditional linear methods. Models were implemented as pipelined models encompassing three steps: feature scaling (excluding DT, RF and XGB), feature selection, and predictive model. See Appendix A, Model Design, for further details on model design, including imbalance handling.

All models were developed based solely on the development cohort dataset. The purpose of this internal validation analysis was solely to conduct model selection, that is, to identify the best model for each target variable for further analysis based on the temporal validation. In particular, the development data was split into a training set (80%) and an internal validation set (20%). The training set was used to perform hyperparameter selection via 5-fold stratified cross-validation, and subsequent re-training on the whole training set. For each task and model class, the configuration of hyperparameters that maximized the F2 score (for the classification task of predicting DD) or R2 score (for the regression tasks of predicting IRLOS and FADL) was selected. Hyperparameters encompassed the feature scaling method, the number of features to be selected, as well as the hyperparameters specific to each class of models.

The full list of hyperparameters is reported in Table 2: for reproducibility purposes, all random seeds were set to 0.

After training, all models were evaluated on the internal validation set in terms of the following statistical metrics: balanced accuracy, sensitivity, specificity, positive predictive value (PPV), negative predictive value (NPV), F1 score, F2 score, Receiver Operating Characteristic Area Under the Curve (ROC AUC), average precision and Brier score (for classification models); absolute error score (AES), mean absolute error (MAE), root mean squared error (RMSE), R2 score (for regression models). ROC curves (for classification models) and error scatter plots (for regression models) were also computed. As the population encompassed both patients who underwent joint replacement at the knee and hip levels, the performance of the models was evaluated not only on the whole internal validation cohort, but also stratified by the main level of intervention. See Appendix A, Stratified Analysis, for further methodological details.

After internal validation, the models were frozen, and the best model for each task was selected. The best model for each task was subsequently validated (without further re-training) on the temporal validation dataset in terms of the same statistical metrics as described above. Similarly to the internal validation, also for the temporal validation, we considered both the overall performance of the best model for each task (that is, the performance on the whole validation cohort) as well as the performance stratified by intervention level (knee/hip).

Additionally, the presence of differences between validation performance was evaluated by means of a statistical hypothesis testing approach [27]. The test was realized as a bootstrap-based [28], one-tailed test. Statistical significance was assessed at the 95% confidence level: that is, a *p*-value lower than 0.05 was considered as indicative of overfitting. See Appendix A, Statistical Analysis, for further details.

To identify the most predictive factors, for each of the three considered tasks, an interpretability analysis of the best developed ML models was also conducted. In particular, feature attributions (also called feature importances) for the best models were computed: for the black-box models, the SHAP post hoc interpretability method [29] was applied to compute feature importances; by contrast, for the transparent models (LiR/LoR and DT), the model (i.e., feature coefficients, for LiR/LoR; decision tree, for DT) itself was analyzed.

All code was implemented in Python (v. 3.13.2) using the following libraries: pandas (v. 2.2.3), matplotlib (v. 3.10.0), scikit-learn (v. 1.6.1), shap (v. 0.47.2), numpy (v. 2.2.4), joblib (v. 1.4.2), scipy (v. 1.15.2), xgboost (v. 3.0.0).

## 3. Results

### 3.1. IRLOS Prediction Task

**Figure 1:**
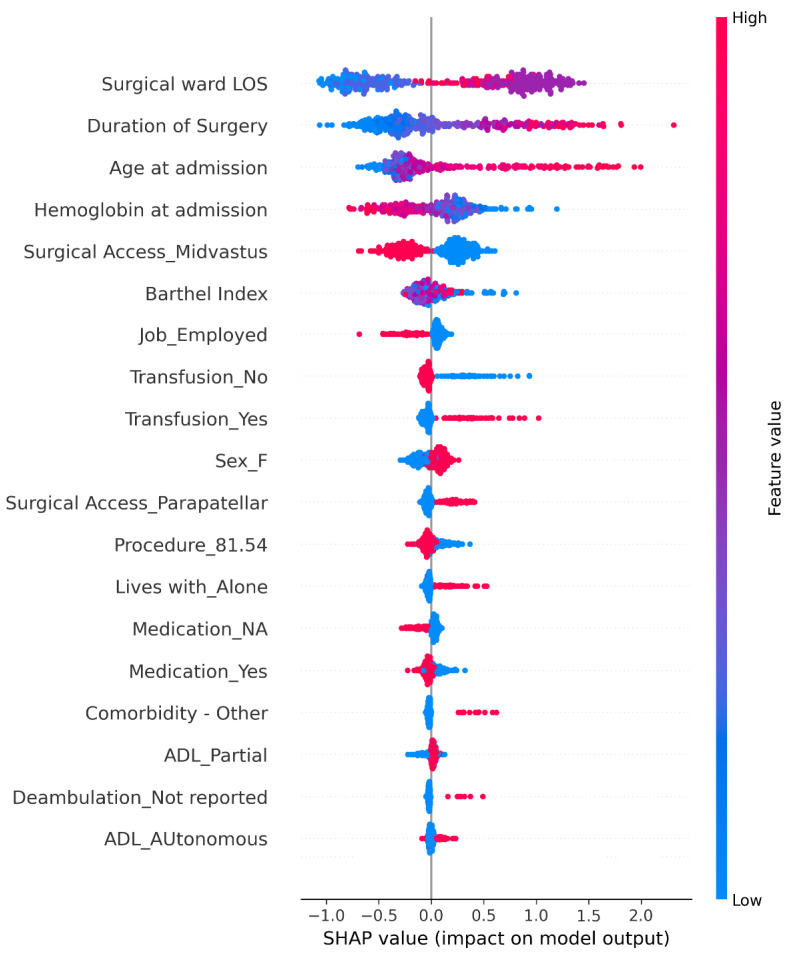
Interpretability analysis for the best model (RF) in the IRLOS prediction task, computed in terms of SHAP values. Each dot represents a prediction: dots on the right of the black vertical line denote an increased IRLOS value. Color denotes the magnitude of the feature values: red denotes higher values, while blue denotes lower values.

The results of the ML models in the internal validation are reported in Table 3 and Figure 1. 

The best model, in terms of all performance metrics, was RF with the following hyperparameters: k = 26, max_depth = 25, n_estimators = 869, max_features = ‘log2’. On the internal validation, for the RF model, no significant difference was detected between the hip and knee sub-cohorts (Bonferroni adjusted *p*-values, AES: 0.100, MAE: 1, RMSE: 1, R2 score: 1). On the temporal validation, the RF model reported an AES of 0.15 (knee: 0.12, hip: 0.19, *p*: 0.300), MAE of 2.52 (knee: 2.53, hip: 2.50, *p*: 1), RMSE of 3.73 (knee: 3.68, hip: 3.83, *p*: 1) and R2 score of 0.17 (knee: 0.14, hip: 0.23, *p*: 1). On the temporal validation, based on the Bonferroni adjusted *p*-values, no significant difference was detected between the hip and knee sub-cohorts. No significant difference (*p*: 0.140) was detected between validation performance in terms of R2 score, though the absolute decrease in performance (Δ = −0.19, −57%) represents a substantial reduction in explained variance.

The results of the interpretability analysis are reported in Figure 1. Surgical ward LOS, surgery duration, age at admission, low hemoglobin, and having received a blood transfusion were the most predictive features for increased IRLOS. By contrast, midvastus access, being employed, and being male were the most predictive features for decreased IRLOS.

### 3.2. FADL Prediction Task

**Figure 2:**
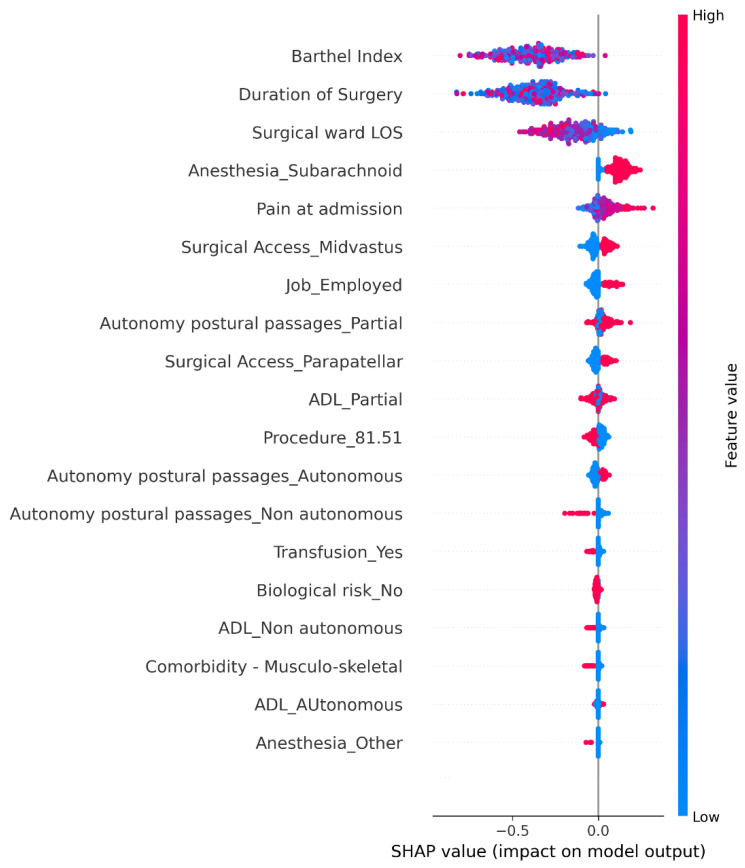
Interpretability analysis for the best model (SVM) in the FADL prediction task, computed in terms of SHAP values. Each dot represents a prediction: dots on the right of the black vertical line denote an increased FADL value. Color denotes the magnitude of the feature values: red denotes higher values, while blue denotes lower values.

The results of the internal validation are reported in Table 4 and Figure 2.

The best model was SVM, with the following hyperparameters: scaler = ‘robust’, k = 20, kernel = ‘rbf’, C = 2.512, epsilon = 0.439, gamma = ‘auto’, max_iter = 1000. On the internal validation, for the SVM model, no significant difference was detected between the hip and knee sub-cohorts (Bonferroni adjusted *p*-values, AES: 0.450, MAE: 1, RMSE: 1, R2: 1). On the temporal validation, the SVM model reported an AES of 0.05 (knee: 0.02, hip: 0.05, *p*: 1), MAE of 2.25 (knee: 2.03, hip: 2.70, *p*: 1), RMSE of 3.04 (knee: 2.71, hip: 3.62, *p*: 1) and R2 score of 0.25 (knee: 0.15, hip: 0.29, *p*: 1). On the temporal validation, based on the Bonferroni adjusted *p*-values, no significant difference was detected between the hip and knee sub-cohorts. No significant difference (*p*: 1.0) was detected between validation performance in terms of R2 score, as the temporal validation performance was greater than the development one (Δ = 0.04, +16%).

The results of the interpretability analysis are reported in Figure 2: lower surgical ward LOS, subarachnoid anesthesia, higher pain at admission, midvastus access, and being employed were the most predictive features for increased FADL. By contrast, a higher Barthel index at baseline and longer duration of surgery were the most predictive features for decreased FADL.

### 3.3. DD Prediction Task

**Figure 3:**
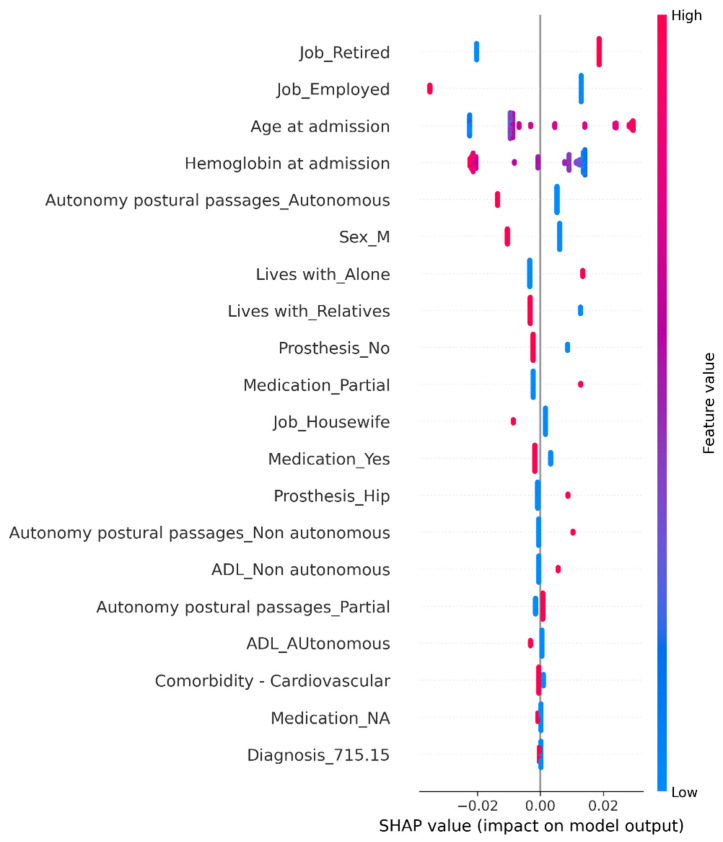
Interpretability analysis for the best model (Random Forest) in the POD prediction task, computed in terms of SHAP values. Each dot represents a prediction: dots on the right of the black vertical line denote an increased risk of discharge at an institutional setting. Color denotes the magnitude of the feature values: red denotes higher values, while blue denotes lower values.

The results of the internal validation are reported in Table 5a (hip and knee arthroplasty combined), Table 5b (knee replacement), Table 5c (hip replacement) and Figure 3.

The best model was RF, with the following hyperparameters: k = 41, criterion = ‘entropy’, max_depth = 1, n_estimators = 965. On the internal validation, for the RF model, significant differences between the hip and knee sub-cohorts were detected only in terms of NPV and Brier score (Bonferroni adjusted *p*-values, accuracy: 0.33, sensitivity: 1, specificity: 0.55, PPV: 1, NPV: <0.001, AUC: 1, F2 score: 1, Brier score: 0, average precision: 1, balanced accuracy: 1). On the temporal validation, the RF model reported an accuracy of 0.69 (knee: 0.72, hip: 0.63, *p*: 0.22), sensitivity of 0.92 (knee: 0.88, hip: 1.00, *p*: 1), specificity of 0.68 (knee: 0.72, hip: 0.61, *p*: 1), PPV of 0.09 (knee: 0.09, hip: 0.10, *p*: 0.330), NPV of 1 (knee: 0.99, hip: 1, *p*: <0.001), AUC of 0.85 (knee: 0.82, hip: 0.88, *p*: 1), F2 score of 0.34 (knee: 0.32, hip: 0.35, *p*: 1), Brier score of 0.22 (knee: 0.22, hip: 0.22, *p*: <0.001), average precision of 0.23 (knee: 0.24, hip: 0.26, *p*: 1) and balanced accuracy of 0.80 (knee: 0.80, hip: 0.81, *p*: 1). On the temporal validation, based on the Bonferroni adjusted *p*-values, significant differences between the hip and knee sub-cohorts were detected only in terms of NPV and Brier score. No significant difference (*p*: 1.0) was detected between internal and temporal validation, though moderate decreases in performance in terms of accuracy, specificity and a small decrease in terms of Brier score were detected (accuracy: −0.06 (−9%), sensitivity: +0.28 (+30%), specificity: −0.09 (−13%), PPV: +0.01 (+11%), NPV: +0.02 (+2%), AUC: +0.14 (+16%), F2: +0.06 (+18%), Brier: −0.01 (−5%), average precision: +0.12 (+52%), balanced accuracy: +0.1 (+12%)).

The results of the interpretability analysis are reported in Figure 3: being retired, older age, being female, or having lower hemoglobin levels at admission were all correlated with increased risk of discharge in an institutional setting. Conversely, not having had a previous implant, living at home (with family members), being autonomous in postural rehabilitation passages, and being employed were all correlated with increased likelihood of discharge in a home setting.

See Appendix B for further results, including the ROC curve and decision curve, as well as the stratified analysis.

## 4. Discussion

### 4.1. ML Feasibility

This study evaluated the predictive performance of ML models for IRLOS, FADL, and DD after total hip or knee replacement, using routinely collected real-world hospital data. The intended application of the models is to support decision-making at rehabilitation admission rather than preoperative risk stratification. Consequently, the inclusion of perioperative variables such as surgical duration and transfusion status does not constitute information leakage but reflects information that would be available in the intended deployment setting. Overall, ML models achieved moderate predictive performance that was stable across intervention type (hip vs. knee) and across time, as assessed by means of temporal validation, while also highlighting clinically relevant predictive features as well as important methodological constraints inherent to real-world clinical prediction tasks.

A recently published scoping review on predictive factors of IRLOS investigated, summarized and illustrated quantitative data from 25 international studies, (a) reporting metadata as publication identifier, year of publication, type of study and data linkage modality; (b) reporting data about country, number of centers involved, setting(s), specific arthroplasty procedure, study population, potential predictors (e.g., clinical, demographic, social, and organizational), actual predictors and outcomes; (c) distinguishing between protocols and completed studies; and (d) including autonomy with the ADL and discharge destination either as variables or as complementary outcomes. None of the 20 completed studies used machine learning techniques. One study on a large population of 7275 patients tested five machine learning algorithms to predict DD following unicompartmental knee arthroplasty, although no validation on another cohort of patients was performed, and inpatient rehabilitation stay was not a necessary part of the pathway [30]. Another study on a population of 5.820 patients tested a machine learning algorithm to predict DD following total joint arthroplasty, and validated the algorithm on a different cohort of patients, although inpatient rehabilitation stay was not the focus of the study [31]. Therefore, our study adds a comprehensive contribution to the literature from many perspectives. IRLOS and FADL, both numerical outcomes, showed modest explanatory power that remained consistent across temporal validation, with temporal validation R^2^ values of 0.17 and 0.25, respectively. These values are consistent with the multifactorial nature of rehabilitation outcomes, which are influenced by a combination of clinical, organizational, behavioral, and social factors, many of which are only partially observed or entirely unmeasured in electronic hospital data. Importantly, performance did not significantly degrade in the validation cohort, supporting the temporal generalizability of the models. We note, however, that in the case of IRLOS, the observed absolute decrease in performance (Δ = −0.19, −57%) represented a substantial reduction in explained variance, which may be clinically relevant: further, more powered validation studies are thus needed to evaluate the generalizability of the IRLOS prediction task.

By contrast, DD prediction, formulated as a binary classification problem, achieved good discrimination (external AUC = 0.85 and balanced accuracy = 0.80) despite a highly imbalanced outcome, while at the same time reporting relatively low positive predictive value, indicating limited ability to accurately identify patients requiring institutional discharge. This pattern reflects the intrinsic difficulty of predicting rare events in real-world cohorts and highlights the importance of selecting evaluation metrics aligned with the intended clinical use. In this context, the high sensitivity and NPV suggest potential usefulness for ruling out patients unlikely to require institutional discharge, rather than for confidently identifying those who will; however, this observation should be considered exploratory and requires formal evaluation of clinical usefulness.

Model performance varied across the outcomes: for both IRLOS and DD prediction, the best model was RF. Being a tree-based ensemble, RF models are intrinsically capable of modeling non-linear interactions and threshold effects (e.g., clinical decisions triggered once certain risk profiles are met), as well as being relatively robust to label imbalance and noise typical of real-world medical data. By contrast, in regard to FADL, the best model was an SVM with an RBF kernel: a possible explanation for this finding may lie in the narrow range taken by Barthel Index values, which is typical of functional scores that often exhibit ceiling effects, as margin-based models (such as SVM) are inherently able to smoothly adapt to small variations in the data while regularizing extreme residuals. More generally, we note that, because the primary objective of this study was prediction rather than parameter estimation, all the outcomes were formulated as supervised learning tasks within a common modeling framework. The total Barthel Index score was modeled as a continuous target despite its ordinal and bounded nature, whereas IRLOS was modeled as a continuous regression outcome despite representing a count of days. Most of the machine-learning algorithms evaluated, including the best-performing models (RBF-kernel SVM for FADL and Random Forest for IRLOS), are nonparametric approaches that do not require the distributional assumptions associated with classical linear or count regression models. Nevertheless, the measurement properties of the Barthel Index and the count nature of IRLOS should be considered when interpreting the results.

In summary, although the predictive performance was modest, such models may provide clinically useful supplementary information to support, rather than replace, clinical judgment. Early estimates of rehabilitation trajectories may assist clinicians in discharge planning, resource allocation, and communication with patients and caregivers. However, the observed predictive performance remains insufficient for autonomous decision-making, and prospective studies are needed to determine whether these predictions improve clinical workflows or patient outcomes.

Previous studies on the prediction of rehabilitation outcomes after total joint arthroplasty have mainly used conventional regression models, with a smaller number of recent works exploring machine learning approaches [11]. Overall, the literature reports heterogeneous and generally moderate predictive performance, reflecting the multifactorial nature of rehabilitation outcomes and the limitations of routinely collected clinical data. Direct comparison between studies remains difficult due to differences in study design, predictors, and validation strategies, with most works relying on internal validation only. In this context, our findings are consistent with previous evidence while extending it through the comparison of multiple machine learning models and the use of temporal external validation.

### 4.2. IRLOS

Several perioperative factors were predictive of longer IRLOS. Surgical ward length of stay emerged as a strong predictor, likely reflecting early complications, delayed mobilization, or medical instability during the acute phase [11,20]. Markers of surgical complexity, such as longer surgery duration, low hemoglobin levels, and the need for blood transfusion, also contributed to prolonged IRLOS, possibly due to increased physiological stress and slower recovery [32]. Age at admission was another relevant factor, consistent with evidence that older patients often present reduced physiological reserve and higher comorbidity burden [11] and lower post-operative function [18]. Conversely, employment status, male sex, and the use of muscle-sparing surgical approaches (e.g., midvastus access) were predictive of shorter IRLOS, possibly reflecting better pre-morbid functional capacity and faster recovery trajectories [33,34]. Social context also played a role, as people living alone often required longer IRLOS before safe discharge.

Overall, these findings support the interpretation of IRLOS as a multidimensional outcome influenced by surgical, medical, functional, and social determinants. From a clinical perspective, early identification of patients at risk for prolonged rehabilitation stay may allow targeted interventions, such as intensified early mobilization, proactive anemia management, and early involvement of social services [35,36].

These findings are broadly consistent with previous studies identifying surgical complexity, age, and early postoperative recovery as major determinants of IRLOS [11,14], while extending previous evidence through a comparative evaluation of multiple ML algorithms and temporal validation.

### 4.3. FADL

Functional autonomy at discharge, measured through the Barthel Index, represents a core outcome of inpatient rehabilitation after joint replacement. In this study, predictive factors for FADL included mainly baseline functional status variables and early perioperative characteristics. Higher baseline Barthel was paradoxically associated with smaller functional gains, a pattern consistent with ceiling effects in rehabilitation outcomes, where people with moderate-to-severe disability may achieve larger improvements [37], and with regression-to-the-mean effects, whereby extreme baseline values tend to be followed by measurements closer to the population average on subsequent assessments.

Shorter surgical ward LOS and the use of subarachnoid anesthesia were predictive of better functional outcomes, likely reflecting smoother perioperative courses and earlier mobilization [11,38]. Interestingly, higher pain at admission was predictive of greater FADL improvement. This may indicate that people with preserved motor function but higher pain levels can achieve substantial functional gains once pain is adequately managed during rehabilitation [39] and may, also in this case, partly reflect regression-to-the-mean effects, as patients presenting with unusually high baseline pain tend to show partial spontaneous reduction over time independent of treatment effects.

Surgical approach and early motor abilities were also relevant. In particular, autonomy in postural transitions emerged as a strong predictor of higher FADL across both hip and knee sub-cohorts, highlighting the importance of early motor assessment [40]. Employment status was likewise predictive of better outcomes, potentially reflecting higher baseline activity levels and motivation toward recovery.

These findings are consistent with previous rehabilitation studies reporting baseline functional status as the strongest predictor of functional recovery after arthroplasty [41].

### 4.4. DD

Discharge destination integrates clinical recovery, functional autonomy, and social context. In this study, institutional discharge was relatively uncommon, but its main predictive factors included age, female sex, and indicators of physiological vulnerability such as low hemoglobin levels [32]. These factors likely reflect reduced functional reserve and increased care needs [42].

Social determinants were particularly influential. Living with relatives and being autonomous in postural transitions increased the likelihood of discharge home, emphasizing the importance of both functional independence and caregiver ability [43]. Conversely, being retired and living alone increased the probability of institutional discharge, reflecting reduced access to informal support networks. Previous implants were also predictive of a lower probability of home discharge, possibly indicating a cumulative functional impairment.

These predictors were largely consistent across hip and knee sub-cohorts, suggesting that discharge planning should focus primarily on global patient characteristics rather than on the type of joint replacement.

The identified predictors are in agreement with previous literature, highlighting that discharge destination depends not only on clinical factors but also on social support and baseline functional independence [21,44].

### 4.5. Limitations

Despite these findings, some limitations should be acknowledged.

First, excluding records with missing values may have introduced selection bias and reduced statistical power. Second, although SHAP-based analyses help interpret model behavior, feature importance should not be interpreted as evidence of causal relationships. Third, the study was conducted in a single center, and local organizational practices may limit generalizability to other healthcare systems. However, the majority of variables are standardized (age, BMI, surgical ward LOS, ASA, duration of surgery, Barthel Index, pain at admission, hemoglobin, diagnoses and procedure codes, sex, biological risk, transfusion, previous prosthesis implanted, and presence of medication) or easily traceable to variables collected in other orthopedic and rehabilitation facilities (comorbidities, surgical access technique, anesthesia, employment, autonomy in the ADL, autonomy in the postural passages, ambulation, caregiver presence, living place, and presence of extra-medication); and the hospital in which the model is trained and validated is a specialized hospital serving a broad population of patients affected by musculoskeletal diseases from the entire country of Italy, which guarantees a substantial degree of usability in routine. Fourth, although temporal validation was performed using an independent cohort from a different calendar year, the validation design does not replicate a prospective implementation scenario, because models were developed using the later cohort and tested on an earlier one. Fifth, while in our performance analysis, we reported the Brier score as a measure of calibration, calibration of the discharge destination model was not formally and more thoroughly assessed; this is particularly relevant given the low prevalence of the positive class and the potential implications for clinical decision-making. The use of ICD-9-CM coding may limit applicability in healthcare systems that have transitioned to ICD-10 or more recent coding standards, although the type of procedure is very common and remains clearly identifiable. Finally, direct quantitative comparison with previous ML studies remains difficult because published models differ substantially in study populations, predictor availability, outcome definitions, and validation strategies.

## 5. Conclusions

This exploratory study demonstrates the feasibility of using machine learning models to predict key inpatient rehabilitation outcomes (length of stay, functional autonomy at discharge, and discharge destination) after total hip or knee replacement using routinely collected real-world hospital data. To our knowledge, this is among the first studies to combine routine hospital data and temporal validation to predict multiple post-acute rehabilitation outcomes after total joint replacement. Although predictive performance was moderate, the models showed stable behavior across intervention type and across time through temporal validation (IRLOS: R^2^ = 0.36 vs. 0.17 (*p*: 0.140); FADL: R^2^ = 0.21 vs. 0.25 (*p*: 1.00), discharge destination: AUC = 0.71 vs. 0.85 (*p*: 1.00), F2: 0.28 vs. 0.34 (*p*: 1.00)), suggesting that they capture relevant patterns in rehabilitation pathways.

The analysis also identified several relevant predictors related to perioperative complexity, baseline functional status, and social context, confirming that rehabilitation outcomes are multidimensional and influenced by both medical and non-medical determinants. From a practical perspective, the developed models may contribute to the future development of CDSSs aimed at supporting clinicians in estimating rehabilitation trajectories and outcomes at the time of admission; however, further validation would be necessary before clinical implementation. Future studies should focus on multicenter external validation, calibration assessment, and prospective evaluation of clinical utility.

## Figures and Tables

**Table 1:** Descriptive statistics of the considered features.

	Mean (Development)	std (Development)	Mean (Temporal)	std (Temporal)
Age at admission	69.19	10.31	69.59	10.36
BMI (Before Surgery)	28.25	5.02	28.62	5.07
Surgical ward LOS	4.78	1.43	5.53	2.16
ASA	1.95	0.35	1.96	0.36
Duration of Surgery	62.5	31.66	71.37	36.09
Barthel Index	70.35	11.96	71.05	10.93
Pain at admission	2.14	1.23	2.28	1.52
Hemoglobin at admission	10.48	1.42	10.44	1.39
Comorbidity	Cardiovascular: 0.68, Respiratory: 0.08, Neuro-cognitive: 0.08, Endocrine: 0.25, Musculoskeletal: 0.07, Tumor: 0.01, Other: 0.05		Cardiovascular: 0.29, Respiratory: 0.06, Neuro-cognitive: 0.17, Endocrine: 0.25, Musculoskeletal: 0.12, Tumor: 0.01, Other: 0.1	
Diagnosis	715.15: 0.34, 715.16: 0.63, Other: 0.03		715.15: 0.32, 715.16: 0.67, Other: 0.01	
Procedure	81.51: 0.36, 81.54: 0.64		81.51: 0.33, 81.54: 0.67	
Surgical Access	Anterior: 0.03, Direct Lateral: 0.01, Midvastus: 0.39, Parapatellar: 0.24, Postero-lateral: 0.33		Anterior: 0.03, Direct Lateral: 0.01, Midvastus: 0.29, Parapatellar: 0.37, Postero-lateral: 0.3	
Sex	F: 0.63, M: 0.37		F: 0.69, M: 0.31	
Job	Housewife: 0.14, Unemployed: 0.06, Employed: 0.2, Retired: 0.6		Housewife: 0.13, Unemployed: 0.05, Employed: 0.2, Retired: 0.63	
Anesthesia	General: 0.04, Subarachnoid: 0.95, Other: 0.01		General: 0.09, Subarachnoid: 0.81, Other: 0.1	
Biological risk	Yes: 0.02, No: 0.98		Yes: 0.01, No: 0.99	
Transfusion	Yes: 0.13, No: 0.87		Yes: 0.11, No: 0.89	
ADL	Autonomous: 0.16, Partial: 0.73, Non-autonomous: 0.11		Autonomous: 0.19, Partial: 0.7, Non-autonomous: 0.11	
Autonomy postural passages	Autonomous: 0.31, Non-autonomous: 0.07, Partial: 0.62		Autonomous: 0.38, Non-autonomous: 0.06, Partial: 0.56	
Deambulation	Crutches: 0.97, Rollator: 0.01, Walker: 0.01, Not reported: 0.02		Crutches: 0.97, Rollator: 0, Walkter: 0.01, Not reported: 0.02	
Lives with	Caregiver: 0.01, Alone: 0.17, Relatives: 0.81, Institution: 0, Other: 0		Caregiver: 0, Alone: 0.24, Relatives: 0.75, Institution: 0, Other: 0	
Prosthesis	No: 0.78, Hip: 0.09, Knee: 0.12, Hip + Knee: 0.01		No: 0.72, Hip: 0.11, Knee: 0.16, Hip + Knee: 0.02	
Medication	Yes: 0.66, No: <0.01, Partial: 0.09, NA: 0.26		Yes: 0.59, No: <0.01, Partial: 0.16, NA: 0.25	
Extra Medication	Yes: 0.25, No: 0.75		Yes: 0.32, No: 0.68	
IRLOS	10.26	3.7	11.67	4.1
FADL	95.7	6.3	97.07	3.51
DD	0.03	0.18	0.05	0.22

**Table 2:** List of tested hyperparameters (*).

	LiR/LoR	SVM	XGB	DT	RF
scaler	[‘min-max’, ‘standard’, ‘yeo-johnson’, ‘max-abs’, ‘normalize’, ‘robust’, None]				
k	Randint (5, num_features)				
penalty	[‘l2’, ‘l1’, ‘elasticnet’]				
max_iter	[10000]	[1000]			
alpha	Uniform (0.001,100)		Uniform (0,5)		
l1_ratio	Uniform (0,1)				
criterion (Regression)				[‘squared_error’, ‘friedman_mse’, ‘absolute_error’, ‘poisson’]	
criterion (Classification)				[‘gini’,’entropy’]	
splitter				[‘best’, ‘random’]	
max_depth			Randint (1,100)	Randint (3,5)	Randint (1,100)
kernel		[‘linear’, ‘rbf’, ‘sigmoid’, ‘poly’]			
C	Uniform (0.001,100)	Uniform (0,10)			
degree		Randint (2,10)			
gamma		[‘auto’, ‘scale’]	Uniform (0,100)		
epsilon		Uniform (0,1)			
n_estimators			Randint (10,1000)		Randint (10,1000)
max_features					[‘sqrt’, ‘log2’]
eta			Uniform (0.01,0.2)		
lambda			Uniform (0,5)		
random_state	[0]				

* Scaler represents the type of feature scaling algorithm applied. K represents the number of selected features. For LiR/LoR: penalty is the regularization loss, max_iter is the maximum number of stochastic gradient descent steps attempted before early stopping, alpha is the regularization coefficient, and l1_ratio is the coefficient associated with the L1 loss (used only if penalty = elasticnet). For SVM: max_iter is the maximum number of optimization steps attempted before early stopping, kernel represents the employed kernel function, C is the regularization coefficient, degree is the polynomial degree (used only if kernel = poly), gamma is the scaling factor (used only if kernel = rbf or sigmoid), and epsilon is the epsilon-insensitivity parameter. For XGB: alpha is the L1 regularization coefficient, max_depth is the maximum depth of the trees in the ensemble, gamma is the label imbalance re-weighting coefficient, n_estimators is the number of trees trained in the ensemble, eta is the learning step, and lambda is the L2 regularization coefficient. For DT: criterion is the splitting criterion, splitter is the split point selection strategy, and max_depth is the maximum depth of the learned tree. For RF: criterion is the splitting criterion, splitter is the split point selection strategy, max_depth is the maximum depth of the trees in the ensemble, n_estimators is the number of trees trained in the ensemble, and max_features is the maximum number of features evaluated at each split. Randint represents a uniform discrete distribution. We refer the interested reader to the scikit-learn API documentation for further information on the hyperparameters.

**Table 3:** Results of the ML models for the IRLOS prediction task (*).

	AES			MAE			RMSE			R2		
	All	Knee	Hip	All	Knee	Hip	All	Knee	Hip	All	Knee	Hip
**LiR**	0.17	0.22	0	2.42	2.3	2.65	3.08	2.95	3.31	0.25	0.32	0.1
**SVM**	0.25	0.28	**0.15**	2.17	2.14	2.34	2.89	2.83	**3**	0.34	0.37	0.26
**RF**	**0.26**	**0.31**	0.12	**2.15**	**2.04**	**2.24**	**2.85**	**2.76**	**3**	**0.36**	**0.4**	**0.27**
**XGB**	0.24	0.28	0.12	2.2	2.13	2.34	2.9	2.84	**3**	0.34	0.37	0.26
**DT**	0.23	0.29	0.06	2.24	2.11	2.48	2.97	2.81	3.24	0.31	0.38	0.14

* For each performance metric, the best model (AES: the higher the better, MAE: the lower the better, RMSE: the lower the better, R2: the higher the better) is highlighted in bold.

**Table 4:** Results of the ML models for the FADL prediction task.

	AES			MAE			RMSE			R2		
	All	Knee	Hip	All	Knee	Hip	All	Knee	Hip	All	Knee	Hip
**LiR**	0.02	0	0.03	2.92	2.72	3.26	5.21	5.04	5.48	0.15	0.15	0.12
**SVM**	**0.15**	**0.17**	0.09	**2.54**	**2.26**	**3.00**	**5.0**	**4.78**	**5.37**	**0.21**	**0.24**	**0.16**
**RF**	0.12	0.11	**0.11**	2.63	2.42	3.05	5.4	4.06	5.96	0.19	0.18	0.02
**XGB**	0.04	0	0.07	2.86	2.72	3.12	5.07	4.89	5.38	0.19	0.20	0.15
**DT**	0.09	0.09	0.07	2.71	2.49	3.12	5.28	5.13	5.53	0.12	0.12	0.10

For each performance metric, the best model (AES: the higher the better, MAE: the lower the better, RMSE: the lower the better, R2: the higher the better) is highlighted in bold.

**Table 5:** (**a**). Results of the ML models for the DD prediction task, hip and knee replacement combined. (**b**). Results of the ML models for the DD prediction task, knee replacement. (**c**). Results of the ML models for the DD prediction task, hip replacement. For each performance metric, the best model (accuracy, sensitivity, specificity, PPV, NPV, AUC, F2, average precision, balanced accuracy: the higher the better, Brier score: the lower the better) is highlighted in bold.

(**a**)										
	**Acc.**	**Sens.**	**Spec.**	**PPV**	**NPV**	**AUC**	**F2**	**Brier**	**Avg. Pr**	**Bal. Acc.**
**LR**	0.67	0.29	0.68	0.03	0.96	0.56	0.11	0.28	0.11	0.48
**SVM**	0.62	**0.64**	0.62	0.06	**0.98**	0.59	0.21	**0.03**	0.05	0.63
**RF**	**0.75**	**0.64**	**0.75**	**0.08**	**0.98**	**0.71**	**0.28**	0.21	**0.11**	**0.7**
**XGB**	0.67	0.5	0.68	0.05	0.97	0.68	0.18	0.2	0.08	0.59
**DT**	0.7	0.57	0.7	0.06	**0.98**	0.67	0.22	0.21	0.06	0.64
(**b**)										
	**Acc.**	**Sens.**	**Spec.**	**PPV**	**NPV**	**AUC**	**F2**	**Brier**	**Avg. Pr**	**Bal. Acc.**
**LR**	0.67	0	0.68	0	0.97	0.43	0	0.28	0.02	0.34
**SVM**	0.6	0.2	0.60	0.01	0.97	0.34	0.04	**0.02**	0.02	0.4
**RF**	**0.74**	**0.62**	**0.75**	**0.03**	**0.99**	**0.69**	**0.26**	0.21	**0.09**	**0.69**
**XGB**	0.69	0.4	0.7	**0.03**	0.98	0.55	0.1	0.19	0.03	0.55
**DT**	0.71	0.40	0.71	**0.03**	0.98	0.5	0.11	0.20	0.03	0.56
(**c**)										
	**Acc.**	**Sens.**	**Spec.**	**PPV**	**NPV**	**AUC**	**F2**	**Brier**	**Avg. Pr**	**Bal. Acc.**
**LR**	0.67	0.44	0.68	0.08	0.95	0.63	0.23	0.28	0.19	0.56
**SVM**	0.68	**0.89**	0.67	0.15	**0.99**	0.75	0.44	**0.06**	0.11	**0.78**
**RF**	**0.77**	0.79	**0.76**	**0.19**	**0.99**	**0.83**	**0.31**	0.21	**0.23**	**0.78**
**XGB**	0.64	0.56	0.64	0.09	0.96	0.75	0.27	0.2	0.17	0.6
**DT**	0.68	0.67	0.68	0.12	0.97	0.75	0.34	0.21	0.13	0.67

**Figure A1:**
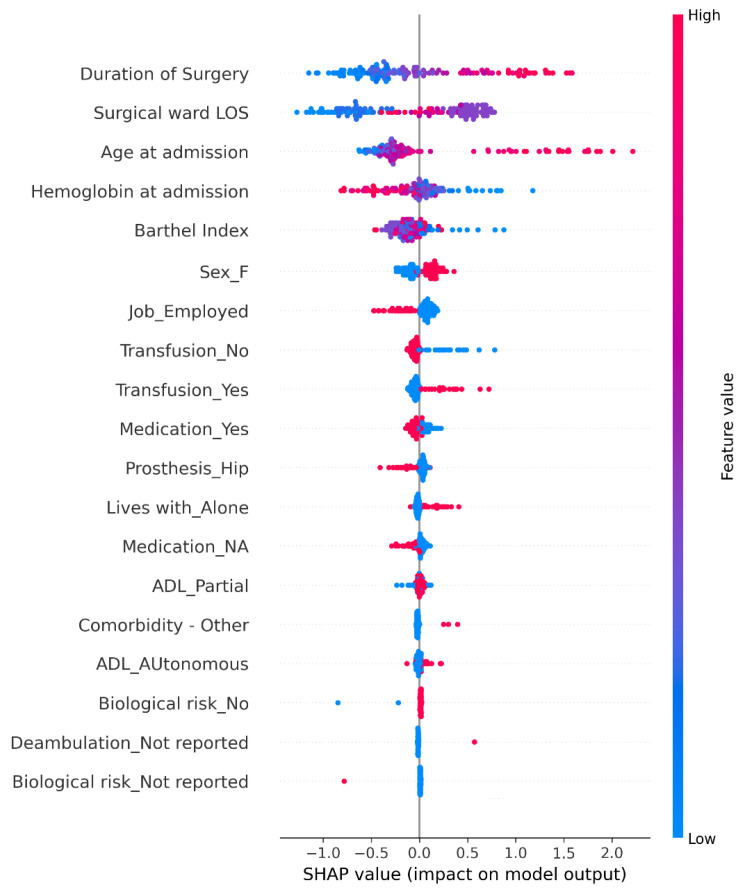
Interpretability analysis for the best model (Random Forest) in the IRLOS prediction task, for the hip sub-cohort, computed in terms of SHAP values. Each dot represents a prediction: dots on the right of the black vertical line denote an increased IRLOS value. Color denotes the magnitude of the feature values: red denotes higher values, while blue denotes lower values.

**Figure A2:**
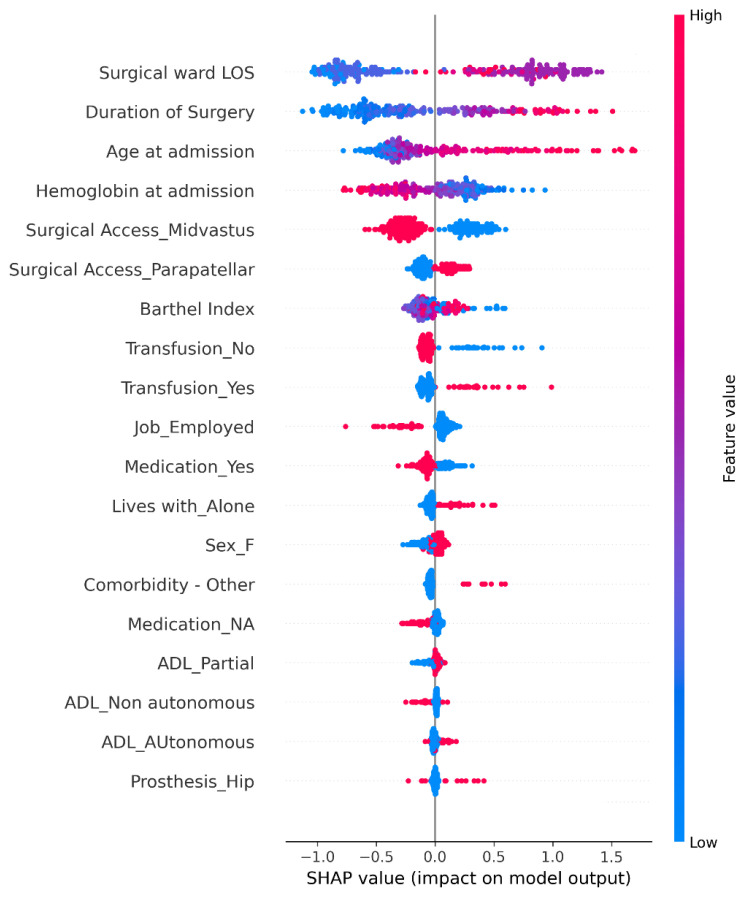
Interpretability analysis for the best model (Random Forest) in the IRLOS prediction task, for the knee sub-cohort, computed in terms of SHAP values. Each dot represents a prediction: dots on the right of the black vertical line denote an increased IRLOS value. Color denotes the magnitude of the feature values: red denotes higher values, while blue denotes lower values.

**Figure A3:**
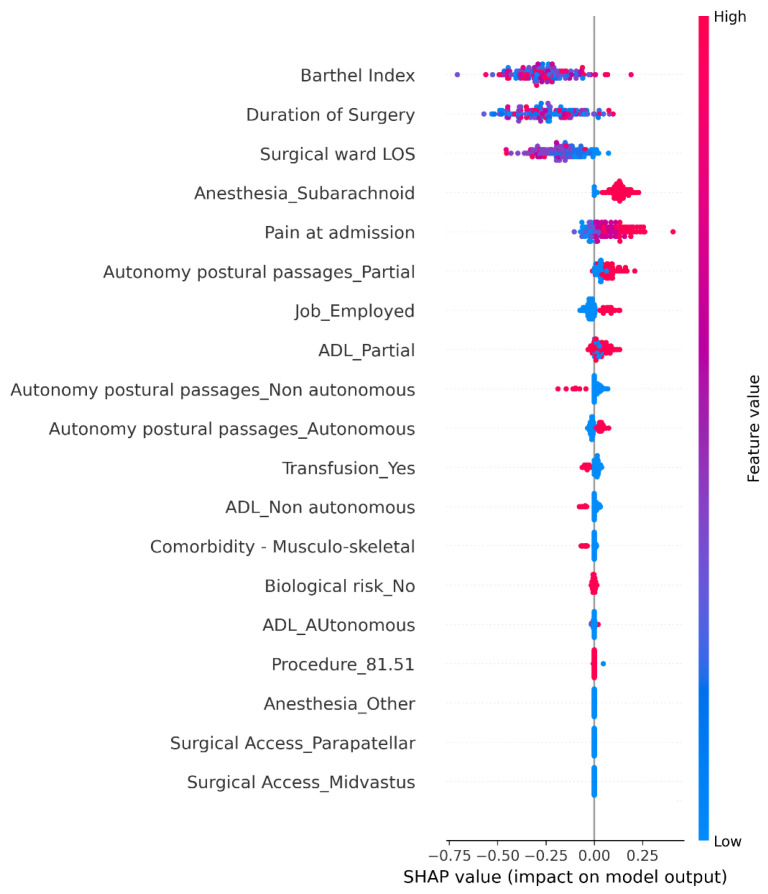
Interpretability analysis for the best model (Random Forest) in the FADL prediction task, for the hip sub-cohort, computed in terms of SHAP values. Each dot represents a prediction: dots on the right of the black vertical line denote an increased FADL value. Color denotes the magnitude of the feature values: red denotes higher values, while blue denotes lower values.

**Figure A4:**
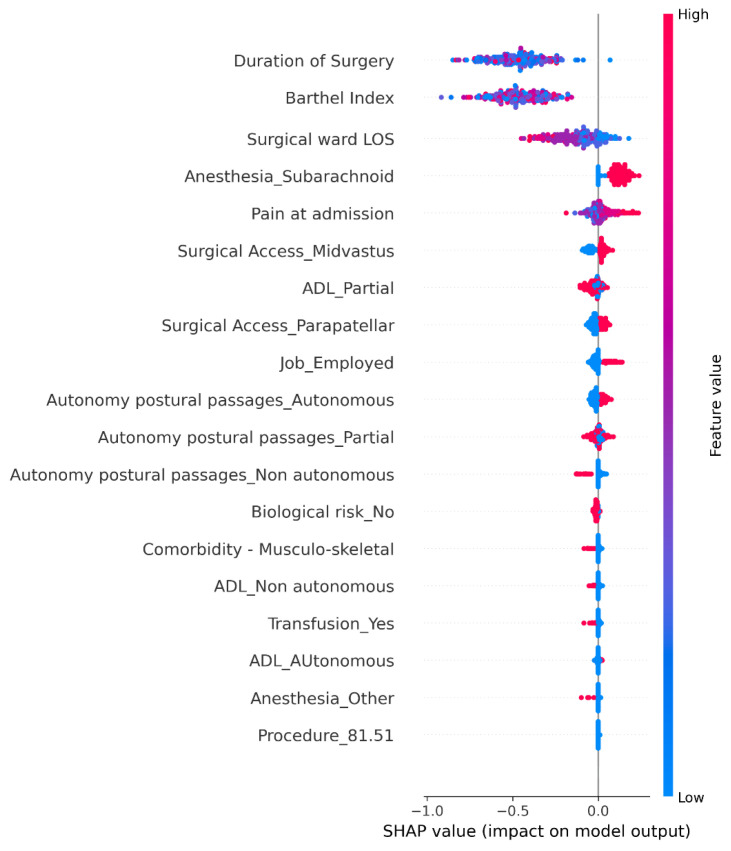
Interpretability analysis for the best model (SVM) in the FADL prediction task, for the knee sub-cohort, computed in terms of SHAP values. Each dot represents a prediction: dots on the right of the black vertical line denote an increased FADL value. Color denotes the magnitude of the feature values: red denotes higher values, while blue denotes lower values.

**Figure A5:**
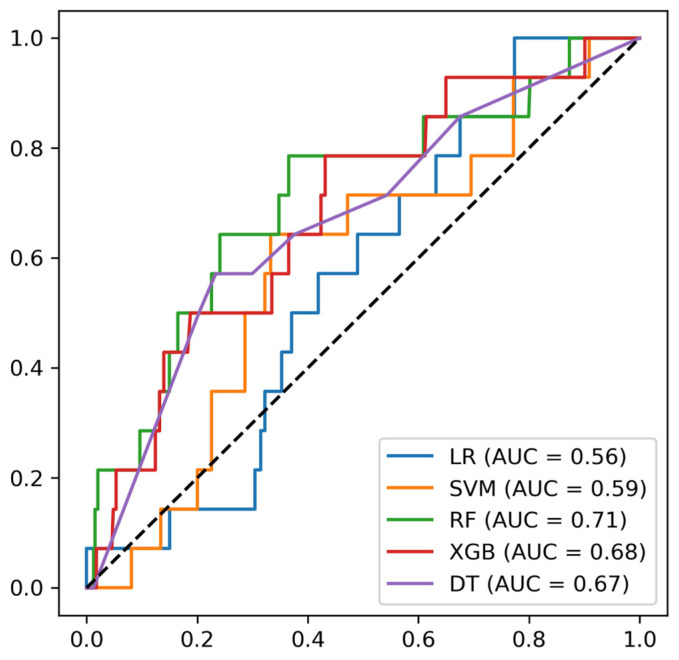
ROC curves, with associated AUC values, for the developed models, calculated on the test set of the development cohort. The dashed line represents the random classifier baseline.

**Figure A6:**
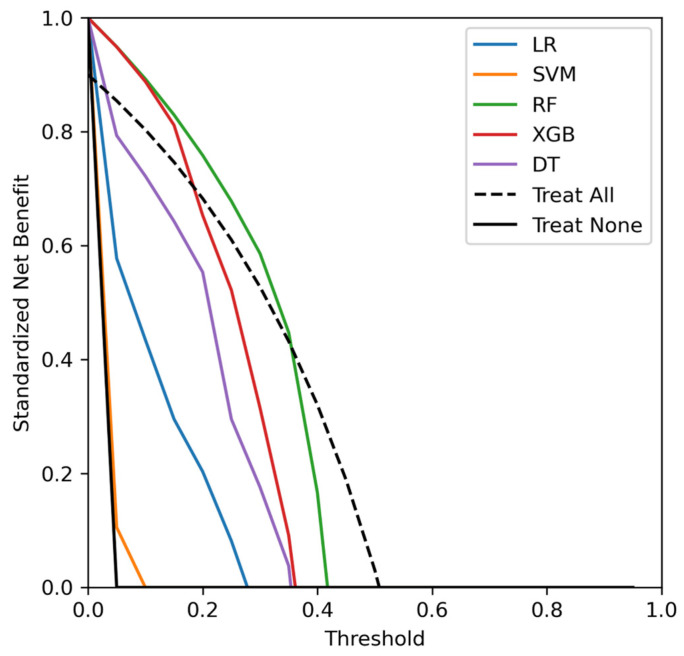
Standardized net benefit curves for the developed models, calculated on the test set of the development cohort. The plot also reports two clinically relevant baselines: treat all (allocate all patients to an institutional setting) and treat none (allocate all patients to a home setting).

## Data Availability

The dataset is available at the following link: https://zenodo.org/records/21104663 (accessed on 14 July 2026).

## Abbreviations

The following abbreviations are used in this manuscript:

RWD	Real-World Data
CDSS(s)	Computerized Clinical Decision-Making Support System(s)
ML	Machine Learning
IRLOS	Inpatient Rehabilitation Length Of Stay
FADL	Function in the Activities of Daily Living
DD	Discharge Destination
ICD-9-CM	International Classification of Diseases—Ninth Revision—Clinical Modification
DT	Decision Tree
SVR	Support Vector Regression
RF	Random Forest Regression
XGB	eXtreme Gradient Boosting
LiR	Linear Regression
LoR	Logistic Regression
ROC AUC	Receiver Operating Characteristics Area Under the Curve
AES	Absolute Error Score
MAE	Mean Absolute Error
RMSE	Root Mean Square Error
PPV	Positive Predictive Value
NPV	Negative Predictive Value

## Appendix A. Supplementary Methods

### Appendix A.1. Data Preparation

Prior to analysis, both the development and temporal validation cohorts’ data were pre-processed to ensure data quality and avoid bias in subsequent steps of the statistical analysis. Pre-existing comorbidities were categorized into seven higher-level groups (cardiovascular, respiratory, neurocognitive, endocrine, musculoskeletal, tumor, among others). Missing values in categorical variables were filled in with a missing indicator value (‘NA’). Subsequently, given the relatively low proportion of missing data (4.6% in both cohorts), a complete-case analysis was performed. To assess the plausibility of the Missing Completely At Random (MCAR) assumption underlying this approach, Little’s MCAR test was conducted separately in the development and temporal validation cohorts: the test did not provide evidence against the MCAR assumption in either the development cohort (*p* = 0.174) or the temporal validation cohort (*p* = 0.956).

Texts were uniformed to avoid typographical errors. Information about the profession of the patients was categorized into four categories (retired, employed, unemployed, and housewife). Similarly, information about the type of anesthesia was grouped into three categories (general, spinal, and other). All the categorical variables were subsequently one-hot-encoded to enable processing with ML models that only handle numerical variables.

### Appendix A.2. Model Design

Feature selection was performed by selecting features with the highest association with the target variable, using mutual information as the association statistic: the number of features to be selected was considered as a hyperparameter to be optimized.

Even though the classification task (DD prediction) was characterized by a significant label imbalance (only 3.5% of the patients were discharged at an institutional setting) only class re-weighting [45] was used as a means of correction: neither under sampling (to avoid discarding data), nor synthetic oversampling (to avoid introducing bias due to the construction of out-of-distribution or meaningless data instances), were applied, in order to maintain the distribution characteristics of the real reference population.

### Appendix A.3. Statistical Analysis

The presence of differences between development and temporal validation performance was evaluated by means of a statistical hypothesis testing approach [27]. The test was realized as a bootstrap-based [28], one-tailed test: the alternative hypothesis was that the models overfitted (i.e., the models performed worse on the temporal validation cohort than on the internal one), against the null hypothesis of no overfitting (i.e., either no difference in performance could be detected, or the models performed better on the temporal validation cohort than on the internal one).

To test the hypothesis, the bootstrap distributions of the models’ internal validation performance (in terms of R2 score for regression tasks, or balanced accuracy for classification tasks) were estimated and used to construct confidence intervals at each level of confidence: the *p*-value was then calculated as the smallest confidence level at which the temporal validation performance of the model was larger than the lower bound of the corresponding confidence interval. Statistical significance was assessed at the 95% confidence level: that is, a *p*-value lower than 0.05 was considered as indicative of overfitting.

### Appendix A.4. Stratified Analysis

Note that the models were trained on the whole cohort and not separately on hip and knee sub-cohorts: this decision ensured a larger training sample while, at the same time, enabling the models to automatically discriminate among the two distinct sub-cohorts. Differences between the two sub-cohorts were assessed based on a bootstrap-based [28] hypothesis test: the null hypothesis was that there was no relevant difference between the two sub-cohorts (i.e., the difference in performance between the two sub-cohorts was neither lower nor higher than for a random stratification of the sample), against the alternative hypothesis of a relevant difference. As differences were evaluated for all the considered performance metrics, the family-wise error rate was controlled by using the Bonferroni correction method [46,47].

## Appendix B. Supplementary Results

### Appendix B.1. IRLOS Prediction Task

In the hip sub-cohort (Figure A1), additionally, low baseline Barthel Index and living alone were identified as predictive features for increased IRLOS, while regularly taking medication as well as having a hip prosthesis were identified as additional predictive features for decreased LOS, with midvastus access not being recognized as predictive in either sense.

Similarly, in the knee sub-cohort (Figure A2), low baseline Barthel Index, parapatellar access, and living alone were identified as additional predictive features for increased IRLOS, while regularly taking medication was identified as an additional predictive feature for decreased IRLOS.

### Appendix B.2. FADL Prediction Task

In the hip sub-cohort (Figure A3), furthermore, being autonomous or partially autonomous in postural passages was identified as an additional predictive feature for increased FADL.

Similarly, in the knee sub-cohort (Figure A4), being autonomous or partially autonomous in postural passages, as well as parapatellar access, were identified as additional predictive features for increased FADL.

### Appendix B.3. DD Prediction Task

In Figure A5 and Figure A6, we report additional details on the performance results in the development cohort, in terms of the receiver operating characteristic (ROC) curve, (standardized) net benefit curve for the considered models.

No relevant differences were detected among the hip and knee sub-cohorts, for which the most predictive features were the same as those detected for the whole population.
